# EIF2B2 gene mutation causing early onset vanishing white matter disease: a case report

**DOI:** 10.1186/s13052-022-01325-3

**Published:** 2022-07-27

**Authors:** Ilaria Filareto, Giulia Cinelli, Ilaria Scalabrini, Elisa Caramaschi, Patrizia Bergonzini, Elisabetta Spezia, Alessandra Todeschini, Lorenzo Iughetti

**Affiliations:** 1 Post Graduate School of Pediatrics, Department of Medical and Surgical Sciences of the Mothers, Children and Adults, University of Modena and Reggio Emilia, Largo del Pozzo, 71 – 41124 Modena, Italy; 2 Department of Medical and Surgical Sciences of the Mothers, Children and Adults, Pediatric Unit, University of Modena and Reggio Emilia, Largo del Pozzo, 71 – 41124 Modena, Italy; 3 Department of Neuroradiology, University Hospital of Modena, Largo del Pozzo, Modena, 71 – 41124 Italy

**Keywords:** Leukoencephalopathy, Vanishing white matter disease, Status epilepticus

## Abstract

**Background:**

Leukoencephalopathy with vanishing white matter (VWM) is an autosomal recessive neurological disease. The physiopathology of disease is still little understood, but it seems to involve impairment in maturation of astrocytes; as a consequence white matter is more prone to cellular stress. Disease is caused by mutations in five genes encoding subunits of the translation initiation factor eIF2B. We know five different types of VWM syndrome classified based different ages of onset (prenatal, infantile, childhood, juvenile and adult onset).

**Case presentation:**

We report the case of a 4-month-old boy with early seizure onset, recurrent hypoglycemia and post mortem diagnosis of vanishing white matter disease (VMD). At the admission he presented suspected critical episodes, resolved after intravenous administration of benzodiazepines. The brain MRI showed total absence of myelination that suggested hypomyelination leukoencephalopathy. The whole exome sequencing (WES) revealed a variant of EIF2B2 gene (p. Val308Met) present in homozygosity. In this case report we also describe the clinical evolution of seizures, in fact the epileptic seizures had a polymorphic aspect, from several complex partial seizures secondarily generalized to status epilepticus.

**Conclusion:**

Infantile and early childhood onset forms are associated with chronic progressive neurological signs, with episodes of rapid neurological worsening, and poor prognosis, with death in few months or years. Clinical presentation of epilepsy is poorly documented and do not include detailed information about the type, time of onset and severity of seizures. No therapeutic strategies for VWM disease have been reported.

## Background

Vanishing white matter disease (VWMD), also known as childhood ataxia with central hypomyelination (CACH disease), is an autosomal recessive leukoencephalopathy. It is caused by mutations in the genes EIF2B1–5 that are involved in initiation and regulation of protein synthesis. VWMD is characterized by a slow progressive deterioration of white matter often exacerbated by infectious episodes or minor head injuries. The typical neuroradiological pattern is characterized by hypo intensity of the white matter in T1 and hyperintensity in T2. Despite being probably the most frequent form of genetic leukoencephalopathy of childhood, onset in the first year of life is very uncommon and associated with rapid decline and poor prognosis in all cases. Here we report a clinical case of a 4 month old boy with early seizure onset and post mortem diagnosis of VWMD.

## Case presentation

A 4-month-old boy born from third-degree consanguineous parents was referred to Pediatric Casualty Ward of Modena’s Hospital for suspected critical episodes, resolved after intravenous administration of benzodiazepines. The episodes were characterized by clones of the right side of the body, chewing movements and eyes blinking; they first appeared a month after a vaccination followed by fever and occurred many times during the day (up to 10), with short duration and self-resolution. A decrement of feeding was also reported as well as loss of previously acquired neuromotor skills with worsening head control and poor reactivity.

The baby was born at 36 weeks of gestation with regular birth history except for transient hypoglycemia; Apgar score 10–10; anthropometric parameters were in the normal range (weight 2950 g, 61° centile; length 49 cm, 67° centile, head circumference 33 cm, 36° centile). The family history was normal.

The neurological examination showed marked axial hypotonus, poor eye contact, social interaction and weak sucking. Vital signs, routine blood exams and electrocardiogram were normal. The body percentiles at the evaluation showed a weight (7160 g) below the third percentile with length (70 cm) and head circumference (34 cm) between 10°-25° percentile.

**Fig. 1:**
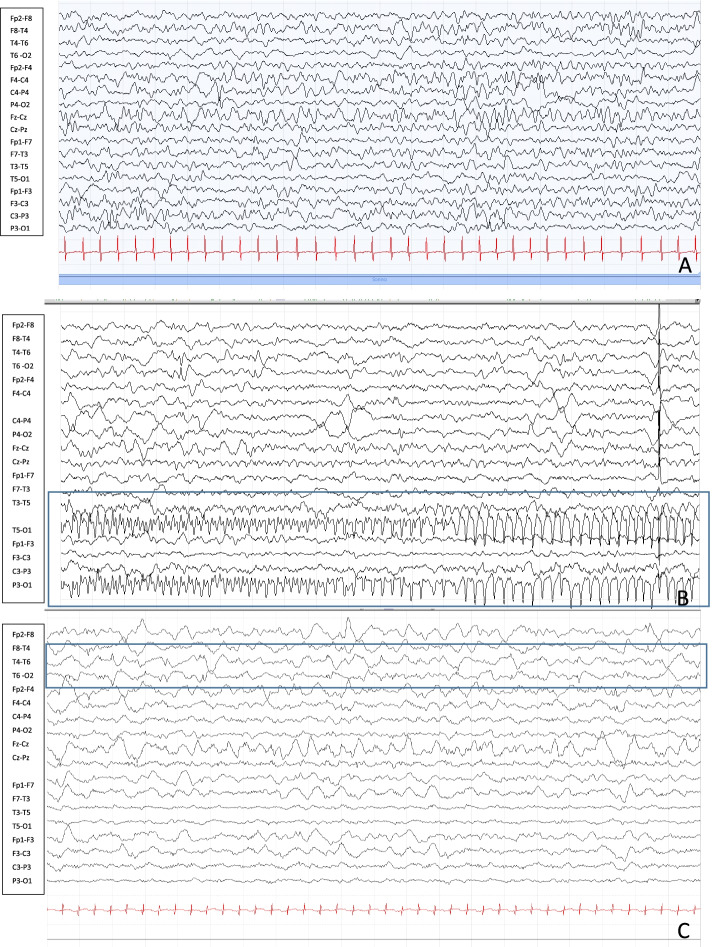
In the Fig. 1 is reported the evolution of the EEG anomalies. At four months of life (**A**) the electroencephalogram reveals recurrent slow waves localized in right temporal hemispheres and asynchronous in the left temporo-occipital area. The EEG at eight months of life (**B**) shows worsening of the background activity by sub continuous presence of focal anomalies, independent over the two hemispheres with recurrent electroclinical episodes characterized by theta-delta activity on the central temporal regions of the right hemisphere and, asynchronously, on the frontal and central regions of the left hemisphere. The EEG at ten months of life (**C**) reveal worsening of underlying activity with electrical disorder and sub-continuous irritant activity especially in in the right temporal zone

The first electroencephalogram (EEG) video registration (Fig. 1A) showed recurrent slow waves localized in right temporal hemispheres and asynchronous in the left temporo-occipital area, sometimes followed by suppression of background activity. During this registration, we tried administration of intravenous pyridoxine (100 mg) without electroclinical improvement. After the beginning of therapy with levetiracetam (57 mg/kg) we observed incomplete response with persistence of 1–2 daily seizures.

**Fig. 2:**
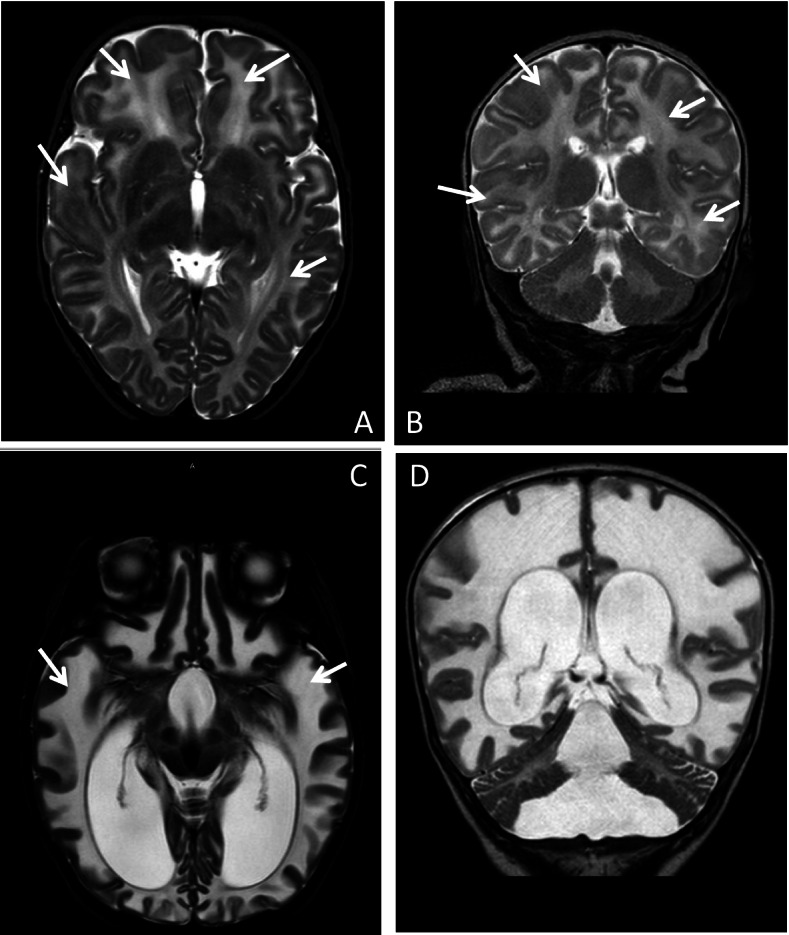
**A** and **B** showed T2 sequences -MRI at three months of life; **C** and **D** were the T2 sequences- MRI performed at ten months of life: in **A** and **B** white matter appears extensively hyperintense with absence of regular myelination. At ten months of life (**C** and **D**) T2-MRI shows increased hyperintensity of both cortical and deep white matter with significant dilatation of the ventricular system

Brain Magnetic Resonance (MRI) was performed and showed thinned corpus callosum, white matter hyperintensity in T2 and hypo intensity in T1, total absence of myelination of the posterior arm of the internal capsule bilaterally (Fig. 2 A and B). The diagnostic orientation was a hypomyelination leukoencephalopathy likely of genetic or metabolic origin.

Complete blood count, liver and renal function test, serum ammonia, and serum lactate were all normal. No alterations of plasma amino acids and urinary organic acids were found and the search for mutated sialotrasferrins was not indicative; metabolic investigations were also negative for oligosaccharidosis, mucopolysaccharidosis, neurotransmitter deficiency, B6 and pyridoxal-phosphate deficiency, primary hyperlactatemia, brain glucose transport deficiency and peroxisomal deficiency. Cardiac and abdominal ultrasound didn’t highlight abnormalities. CGH array did not document anomalies such as duplications or deletions.

During the following months the infant was hospitalized several times for the worsening of seizures, often triggered by infectious episodes. Several trials with phenytoin, phenobarbital e diazepam have been made, but the association of levetiracetam, clonazepam and ketogenic diet gave better results. ACTH has been administrated with only partial response.

In the following months the patient needed numerous hospitalizations for worsening of the epileptic seizures, treated with continuous infusion and boluses of intravenous midazolam. In this period in the epileptic seizures had a polymorphic aspect, we observed several complex partial seizures secondarily generalized with status epilepticus and need for intensive support. In the intercritical period we observed numerous episodes, characterized by clones of limbs, deviation of the eyes, drooling and masticatory movements lasting a few seconds with a frequency between five and ten episodes in an hour.

The clinical history was also characterized by frequent episodes of hypoglycemia during a ketogenic diet despite ketonemia values within the therapeutic limits (ketonemia between 2 and 5 mmol/L). The endocrine and metabolic tests performed were not indicative, immunodeficiency was excluded. The EEG evolution showed, at eight months of age, a marked worsening of the background activity with sub continuous presence of focal abnormalities like spike and slow high voltage waves, independent over the two hemispheres; we could observe recurrent electroclinical episodes characterized by theta-delta activity on the central temporal regions of the right hemisphere and, asynchronously, on the frontal and central regions of the left hemisphere (Fig. 1 B); in correspondence of these episodes the patient showed repetitive head movements, deviation of eyes towards left side, eyelids clones, nystagmus, uncoordinated diaphragm movements and right wrist writing movements with migrating clones from right to left side of the body in absence of hyper tone and clones or myoclonic events.

At ten month of life, the EEG with video-registration showed further worsening of underlying activity with electrical disorder and sub-continuous irritant activity of slow high voltage waves especially in in the right temporal zone (Fig. 1 C).

The patient was hospitalized one last time at one year of life for sub-continuous epileptic episodes associated with bradycardia and desaturation that led to degeneration of general clinical conditions and then to death.

**Fig. 3:**
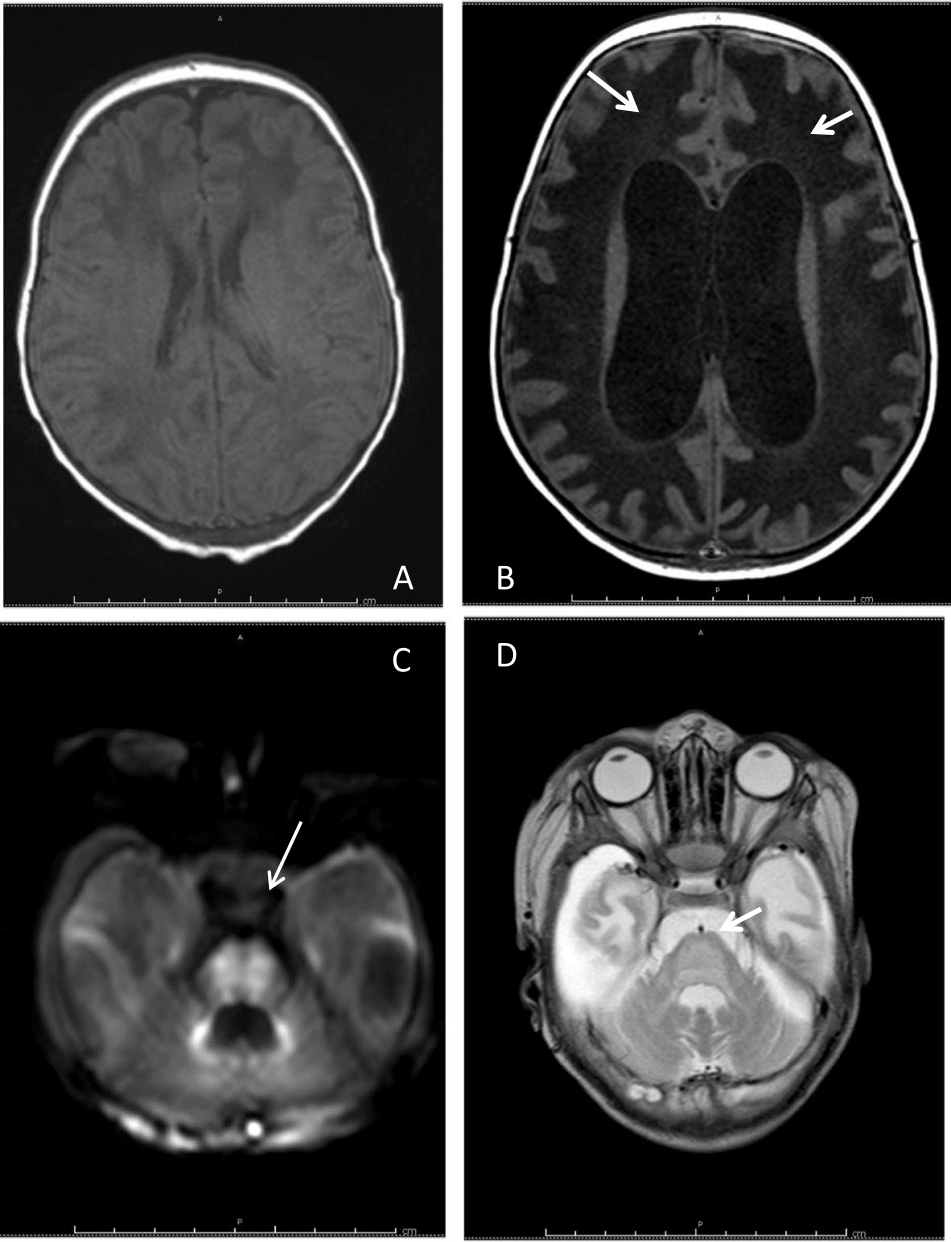
**A** shows T1 sequences -MRI at three months of life; **B** represents the T1 sequences- MRI performed at ten months of life. Both images reveal hypointensity of white matter in T1 sequences. **C** e **D** were performed before death and showed alteration of the signal and complete involvement of the bulb

During the observation we repeated MRI that showed a marked reduction of white matter not only in the cerebral hemispheres but also in midbrain with almost complete involvement of the bulb. It was associated to important dilatation of the supra and sub tentorial ventricular system (Fig. 3 C and D). The diagnostic suspect, based on clinic course and imaging, was VWMD, confirmed post-mortem by clinic exome. The analysis revealed a variant in homozygosity (p.Val308Met) of EIF2B2 gene. This mutation is pathognomonic of VWMD and the parents were carriers in heterozygosity. Furthermore, post mortem results were obtained from the investigations on the respiratory chains which documented the reduction of the activity of complexes I and IV on muscle. Exome analysis revealed no mutations in nuclear genes associated with mitochondrial diseases.

## Discussion and conclusion

VWMD or childhood ataxia with central hypomyelination (CACH) syndrome is an autosomal recessive leukoencephalopathy. VWM is caused by mutations in the genes EIF2B1-5, encoding the subunits of eIF2B, a protein necessary for translation initiation and regulation of protein synthesis under different conditions, including cellular stress [1]. The prevalence of known living affected individuals is ~ 1.4:1,000,000 inhabitants, while the prevalence of individuals carrying two mutant alleles of a gene encoding a specific eIF2B subunit has been estimated to be 1:80,000–100,000 live births [1]. It is a chronic progressive disease with episodes of rapid and major neurological worsening caused by stress (fever, minor head trauma, acute fright) with incomplete recovery [2, 3].

The physiopathology of the disease is still little understood, but it seems to involve impairment in maturation of astrocytes; as a consequence the white matter is more prone to cellular stress. No effective therapy is known. Corticosteroids have proven to be sometimes useful in acute stages [4].

We know five different types of VWM syndrome classified based different age of onset (prenatal, infantile, childhood, juvenile and adult onset). The earlier is the age of onset, the higher is the risk of rapid evolution and death [5]. The childhood VWMD occurs primarily between age 2 to 6 years [6], while later/adult onset VWMD with either clinical manifestations or MRI abnormality alone is also recognized and usually implies a milder disease course [7]. The infantile VWMD variant is typically characterized by rapid neurological deterioration, ataxia and spasticity.

In a follow up study that involved 14 infantile and 26 childhood patients Zhou et al. [8] compared the natural history and the typical distribution of the damage in Childhood and Infantile VWMD. Compared with early childhood onset VWMD, infantile VWMD showed more extensive involvement of the subcortical WM in the frontal lobe, anterior limb of the internal capsule, midbrain, pons and dentate nuclei of the cerebellum. As a matter of fact, the second RMI performed to our patient showed marked worsening of white matter impairment extended to cerebral hemispheres, pons, midbrain and bulb.

MRI is important for differential diagnosis; indeed in patients presenting with rapid neurological deterioration it is important to exclude demyelinating encephalomyelitis and encephalitis. In patients with subacute or chronic neurological deterioration and an MRI showing diffuse cerebral white matter abnormalities, mitochondrial leukoencephalopathies are important disorders in the differential diagnosis. In mitochondrial defects, MRI typically shows well delineated cysts, the white-matter abnormalities tend to be less diffuse then in the VWM [9].

White matter abnormalities similar to VMD are described in metabolic disease such as L-2-hydroxyglutaric aciduria, non-ketotic hyperglycinemia, classical phenylketonuria, mucopolysaccharidoses, GM gangliosidoses, Zellweger disease, adrenomyeloneuropathy and Van der Knaap disease [10].

Infantile and childhood patients shared similarities in the incidence of epileptic seizure (35.7 vs. 38.5%) and episodic aggravation (92.9 vs. 84.6%). Developmental delay before disease onset was more common in infantile patients. The distribution of mutations among EIF2B1—5 was not significantly different between infantile and early childhood patients. [8]

Although correlation between genotype and phenotype is not described in literature, it has been observed that mutations in not conserved amino acids are associated with later onset, slow progression and longer survival, giving the impression that a specific genetic mutation might predict a specific clinical phenotype [11]. This observation is consistent with our experience, because the residual amino acid was highly conserved and we observed early onset and rapid worsening.

This report shows a rare case of VWMD syndrome with onset at four months of age. As mentioned before, case reports of infantile forms of VWMD are very infrequent. The diagnosis was confirmed by post-mortem diagnostics through clinical exome analysis documenting mutation in homozygosis of the EIF2B2 gene (p.Val308Met). The analysis in parents revealed the presence of this mutation in heterozygosis. This variant is very rare in the general population (0.000008122) and described by Zhang et al. [12] in 2015 in heterozygosis with another mutation, while Rezaei et al. [13] described the same mutation in homozygosis.

In a recent proteomics study focusing on remyelination in adult mice brains in response to cuprizone-induced demyelination, Gat Viks et al. found that dysregulation of mitochondrial functions, altered proteasomal activity and impaired balance between protein synthesis and degradation play a role in VWM pathology [14]. Since post-mortem brains of several VWMD patients contain oligodendrocytes with “foamy” cytoplasm due to increased number of abnormal mitochondrial membranes [15], we aimed to find a biochemical link between eIF2B mutations and mitochondrial function. A study by Raini et al. in 2016 [16] on mouse embryonic fibroblasts (MEFs) showed decreased activity of complex I and complex IV by 30% and 40%, respectively, in mutated MEFs compared to wild type. A similar result was found in our patient’s muscular biopsy.

Recent studies have shown a rare association between VWMD and hyperinsulinemia hypoglycemia. Bursle et al. reported three cases of infantile onset VWMD with hypoglycemia [17]. In these patients hypoglycemia became apparent at 6 and 8 months of life, although in one patient transient was also documented neonatal hypoglycemia.

The pathophysiology of hyperinsulinism in VWMD is not clear; it may involve dysregulation of transcription of genes related to insulin secretion. We have described a rare case of infantile VWMD with rapid evolution and association with several episodes of hypoglycemia. In literature are reported few cases of this association, unfortunately these patients have invariably a bad prognosis.

In Table 1 we illustrated all cases of infantile VWMD reported in literature and we observed that the most frequent forms of early onset VWMD are linked to mutation of EIF2B5. Clinical phenotype is variable and the most common manifestations are worsening and progressive psychomotor deterioration, hypotonia, epilepsy and feeding difficulties. Epilepsy is common in these patients but forms of refractory epilepsy are not frequently reported in early onset VWMD. The case described by Rezaei et al. is also characterized by early-onset seizures refractory to antiepileptic drugs. In this case a good control was obtained with the association of phenobarbital, topiramate, lamotrigine, and oxcarbazepine [13].

**Table 1:** Pediatric case reports on vanishing white matter disease with onset in the first two years of lives

AUTHOR /YEAR	ONSET AGE	TRIGGER	SYMPTOMS	SEIZURES	MRI	MUTATION	OUTCOMES
**FRANCALANCI ET AL (2001)** [18]	11-mo (oldest sister)	Upper respiratory tract infection	Loss of motor abilities, nystagmus, tetraplegia, intermittent dystonia, opisthotonus, seizures, and feeding difficulties	Not described	Generalized hypo-intensity of the white matter in T1-weighted images, which turned hyperintense in T2-weighted section	EIF2B5- 3q27 cr	Death at 18-mo
**ROSEMBERG ET AL (2002) ** [19]	15- mo	Not reported, first investigation after brother’s death	Worsening in psychomotor development from 2-mo, progressive psychomotor deterioration, generalized seizures	Generalized seizures	Diffuse involvement of hemispheric and cerebellar white matter, external capsules, corpus callosum and pons	UK	At 2 years 8 months of age bedridden without contact with surroundings
**ROSEMBERG ET AL (2002) ** [19]	8-mo	Not reported	Hypotonia, loss of head control, hypertonia of limbs	EEG showed generalized slow waves, seizures not described	Diffuse involvement of the supratentorial white matter hypointensity on T1-weighted images and hyperintensity on T2-weighted images	UK	Progression of hypertonia, loss of visual contact, deceleration of head circumference in the next 2 years
**SIJENS PE ET AL (2005)** [20]	15-mo	Febrile illness with vomiting, worsening after head injury	Ataxia, spasticity, seizures	Not described	hypointensity of cerebral white matter	UK	Not reported
**SHARMA ET AL (2014)** [21]	18-mo	Febrile illness 5-mo before	Spasticity of limbs with dystonic posture, regression of milestones	Absent	Extensive white matter rarefaction in FLAIR images involving posterior limb of internal capsule, corpus callosum and cerebellar white matter	EIF2B5 compound heterozygous mutation	Not reported
**HATA ET AL (2014) ** [22]	10-mo	Not reported	Seizures, Physical and mental deterioration,	clonic convulsions with facials twitches	progressive dilatation of the lateral ventricles and vanishing white matter	EIF2B2	Death at 4 years old
**TAKANO ET AL (2015) ** [23]	4-mo	Vaccination three days before	Intractable seizures, lethargy, vomiting, rapid neurological deterioration	Refractory complex-partial seizures, several status epilepticus, EEG with high voltage multifocal spikes	Diffuse bilateral symmetrical abnormal signal of the deep WM characterized by a FLAIR hypointensity	EIF2B5 compound heterozygous mutation	Bedridden, spastic quadriplegic, home oxygen therapy and nasoduodenal feeding at 25-mo
**GUNGOR ET AL (2015)** [24]	3-mo	Not reported	Refractory generalized tonic, myoclonic seizures, psychomotor development deteriorated	EEG with voltage suppression in both hemispheres and sharp spike-slow-wave complexes in the right hemisphere	cerebral and cerebellar WM involvement with progression to cerebellar atrophy and cystic degenerations in cerebral WM	EIF2B4 homozygous mutation	severe mental and motor retardation after 8-mo
**ESMER ET AL (2017)** [25]	13-mo	Fever illness	Hypotonia, drowsiness, focal seizures, cognitive and motor deterioration	Not described	Generalized WM hypointensity	EIF2B5 Homozygous mutation exon 2	Death at six years due to respiratory failure
**SONG ET AL (2017) ** [26]	4-mo (older brother)	Finding of prenatal ventriculomegaly	Generalized seizures, poor head control, severe extremity spasticity with scissoring lower-extremity posture and hyperreflexia	Generalized seizures, no more description	First performed at 3-mo: diffusely abnormal cerebral WM signal with antero-temporal and parieto-occipital cysts	EIF2B3 Homozygous mutation	neurologic status declined, death at 8.5 months of respiratory arrest
**SONG ET AL (2017) ** [26]	Prenatal (younger brother)	Prenatal bilateral ventriculomegaly and intrauterin growth retardation	spasticity, encephalopathy	Not reported	Fetal MRI: diffusely abnormal cerebral WM	EIF2B3 Homozygous mutation	failure to thrive and death by 8 months of age from respiratory arrest
**PORCIUNCULA ET AL (2018)** [27]	5-mo	Illness fever with conjunctivitis two day before	Refuse breastfeeding, lethargy, hypotonia, seizures, neurological regression	Tonic–clonic refractory seizures with masticatory movements,	Abnormal diffuse and symmetric signal affecting the WM	EIF2B5 homozygous mutation	Coma state after acute infection with epileptic state ending with death at 11-mo
**REZAEI ET AL (2018) ** [13]	4-mo	Not reported	Poor weight gain, refractory epilepsy, hypotonia, microcephaly, dense cataract, global developmental delay	frequent seizures with staring, clonic movements of the left side limbs and truncal hypotonia	Abnormal diffuse signal affecting the WM with early cistic degeneration	EIF2B2 homozygous mutation	Seizures under control at 12 months with multiple AED, home oxygen therapy and mechanical ventilation throught T-tube
**OUR PATIENT**	2-mo	Vaccination, viral infections	Epilepsy, development deteriorated, feeding difficulties	complex partial seizures secondarily generalized, masticatory movements	Reduction of WM in the cerebral hemispheres, in ponto-mensencephalic tegmentum and complete involvement of the bulb	EIF2B2 homozygous mutation	Death

Although refractory generalized tonic- myoclonic seizures were previously described [23, 24, 27], clinical presentation of epilepsy is poorly documented and do not include detailed information about the type, time of onset and severity of seizures. Most of patients reported in Table 1 present conditions of stress as a trigger of rapid and major neurological worsening [18, 20, 21, 23, 25].

In conclusion we described a case of early onset VWMD rapid evolution and association with refractory epilepsy and several episodes of hypoglycemia. This report shows also the clinical evolution of seizures, the onset and role of stress condition (fever, head trauma, viral infection) in neurological worsening. The treatment is based on antiepileptic drugs and support care. Clinicians play a key role in the management of these patients, because no therapeutic strategies for VWMD disease are reported.

## Data Availability

The datasets used and analyzed during the current study are available from the corresponding author on reasonable request.

## Abbreviations

VWMD: Vanishing white matter disease
eIF2B: Eukaryotic initiation factor 2B
WES: Whole exome sequencing
EEG: Electroencephalogram
MRI: Magnetic Resonance imaging
CACH: Childhood ataxia with central hypomyelination
VWM: Vanishing white matter
WM: White matter
MEFs: Mouse embryonic fibroblasts
